# Improving *Telenomus remus* (Hymenoptera: Scelionidae) Adoption: Contribution of Different Egg Parasitoid Densities, Fed Adults, and Their Storage for Successful Biological Control of *Spodoptera frugiperda* (Lepidoptera: Noctuidae)

**DOI:** 10.3390/insects16101032

**Published:** 2025-10-06

**Authors:** Weidson P. Sutil, Adeney de F. Bueno, Leonardo Roswadoski, Rafael S. Iasczczaki, Gabriel S. Carneiro, Yelitza C. Colmenarez

**Affiliations:** 1 Department of Biology, Federal University of Paraná, Curitiba 81531-980, PR, Brazil; plauter80@gmail.com (W.P.S.); leonardoroswadoski@ufpr.br (L.R.); gabriel.siqueira@colaborador.embrapa.br (G.S.C.); 2 Embrapa Soybean, Caixa Postal 4006, Londrina 86085-981, PR, Brazil; 3 Department of Entomology, Federal University of Viçosa, Viçosa 36570-900, MG, Brazil; rafael.iasczczaki@ufv.br; 4 CABI Latin America, Foundation of Agricultural and Forestry Studies and Research (FEPAF)—Avenida Universitária, Botucatu 18610-034, SP, Brazil; y.colmenarez@cabi.org

**Keywords:** shelf live, parasitoid nutrition, fall armyworm, noctuids

## Abstract

Egg parasitoids, such as *Telenomus remus* (Hymenoptera: Scelionidae), face significant challenges after release, as their pupae are exposed to various mortality factors that reduce the efficiency of biological control programs. Therefore, this study aimed to evaluate a honey-solid diet that can feed adults still inside the capsules without sticking the wasps on its surface, enabling parasitoid storage and later field release. Three independent bioassays were performed, each with 20 completely randomized replications. The first bioassay evaluated the acceptance of a solid feed—honey soaked in cotton thread—compared to the traditional form—honey droplets. In the second bioassay, the storage periods after emergence of adults in capsules with honey-solid food were analyzed at 2, 4, 6, and 8 days post-emergence, and the third bioassay studied the efficacy of different release densities of fed adults under field conditions. Parasitoids fed on the honey-solid diet exhibited a 13.3% reduction in parasitism compared to honey droplets. However, the sticky, viscous nature of honey can lead to parasitoids becoming glued, potentially leading to their death. *T. remus* feeding on the honey-solid diet resulted in low mortality inside the capsules, living up to six days with only 22.2% reduction in parasitism capacity, making it a viable alternative to release and transport fed adult parasitoids, with an increase of around 30% in the released density of parasitoids compared with the parasitoids fed on honey droplets. This flexibility of releasing *T. remus* up to six days after emergence provided valuable knowledge to establish *T. remus* as a biocontrol agent. Furthermore, the highest tested parasitoid density of 20,000 parasitoids per hectare obtained the highest parasitism of *Spodoptera frugiperda* (Lepidoptera: Noctuidae) eggs. However, future studies are still required with higher releasing densities and less expensive methods of mass rearing the parasitoid for those higher densities to be economically viable.

## Simple Summary

Employing beneficial insects to control crop pests is a sustainable alternative to chemical pesticides, but some important questions remain for their successful use in the field. For instance, how many *Telenomus remus* (Hymenoptera: Scelionidae) should be released to effectively reduce *Spodoptera frugiperda* (Lepidoptera: Noctuidae) populations, and how to keep them alive and healthy before release remains unclear. *T. remus* is a tiny wasp that helps control *S. frugiperda* by attacking its eggs, but many individuals die before reaching the field, reducing the impact of biological control. This study tested a solidified food source that can be placed inside the release capsules to feed the parasitoids before their release. The results showed that the insects accepted this food well, allowing them to be stored for up to six days inside the capsules without major biological parameter costs. We also tested different release densities and found that even the highest density studied (20,000/ha) is not sufficient to effectively control *S. frugiperda* by itself. These findings help improve how this parasitoid is used for biological control and support the development of more effective and environmentally friendly *S. frugiperda* management strategies.

## 1. Introduction

Fall armyworm (FAW) *Spodoptera frugiperda* (Smith) (Lepidoptera: Noctuidae) is a pest native to the New World [1]. As a highly polyphagous species, FAW larvae feed on leaves, stems, and reproductive structures of 353 host plants belonging to 76 botanical families [2] and is a key pest of several important staple crops [1], especially maize [3]. Although genetically modified maize varieties can provide excellent control of FAW [4], insecticides are still widely adopted due to increased cases of resistance [5,6,7]. However, the overuse of chemicals has raised global concerns about their negative side-effects on the environment and human health [8,9]. Those concerns have gained increasing attention, especially due to the extension of area in which FAW occur, invading new regions such as Africa, Asia, Australia, and Europe [10,11]. This has made FAW a global threat to food security [12] increasing the demands for more sustainable tools for its management [13].

Biological control is among the most eco-friendly alternatives against FAW [14]. Egg parasitoids are noteworthy biocontrol agents for controlling the pest in its first stage (egg) before any damage to its host plants (crops) [15]. Several different species of the genus *Telenomus*, for instance *Telenomus remus* (Nixon) and *Telenomus dignus* (Gahan), have been reported as eggs parasitoids of FAW [16]. *T. remus* has emerged as one of the most prominent and widely reported candidates for Augmentative Biological Control (ABC) programs of FAW around the world [17,18,19]. It stands out [20] due to its high parasitism capacity on *Spodoptera* spp. eggs [18], the high dispersal capacity of its adults [21], and great host foraging potential [22]. Furthermore, its adults are able to parasitize *Spodoptera* eggs on overlapping layers; including those eggs located in the inner layers of egg masses, frequently covered with scales from the moth’s wings [19]. Due to this extraordinary potential, *T. remus* has been one of the most studied egg parasitoids against *Spodoptera* spp., including FAW, in different countries around the world, especially in Latin America [18,23,24,25,26,27,28].

The first releases of *T. remus* in Latin America were performed manually in the 1990s on small scales with overall positive results [18]. Releases of *T. remus* in Venezuela, Colombia, Guyana, and Suriname achieved up to 80–100% FAW egg parasitism [29,30,31,32,33]. To scale up the adoption of egg parasitoids as a biological control strategy over large areas, the biological control industry has preferred to release pupae close to adult emergence, due to their easy mechanization of the release process [34]. However, males of *T. remus* emerge up to 24 h earlier than females [35]. Therefore, *T. remus* females released as pupae will stay longer in the field before adult emergence. This makes the released parasitoid, especially females, extremely vulnerable to different causes of mortality. High temperatures [36,37,38], rainfall [39], and predation [40] are the most frequent and have the greatest impact on parasitoid survival in the field immediately after release. Therefore, evaluating new strategies to release *T. remus* in field conditions has great theoretical and practical interest [18]. The refinement of protocols to release biocontrol agents, for instance, *T. remus*, in ABC programs is one of the major challenges to improving the use of biocontrol strategies within integrated pest management (IPM) [41].

Despite the increasing interest in *T. remus* for biological control in Brazil, where it has only recently been officially registered for commercial use, key operational parameters still require study. In South America, release densities of *T. remus* in fields of maize vary widely from 5000 to 200,000 parasitoids per hectare [31,32,42,43,44,45,46,47,48,49,50]. This undefined recommendation protocol of how to release *T. remus* could be responsible for the variations in the results recorded in the literature. Some Brazilian studies [47,50] reported lower parasitism (from 1.4 to 9.5%) after releasing *T. remus* (≤24 h old) following single releases of 100,000–200,000 individuals per hectare at the emergence or the first parasitoids. However, considering the males emerge up to 24 h before females [15], the released parasitoids were probably mostly males, and the remaining pupae (females) were subjected to significant mortality factors for longer periods in the field [18]. Adverse temperature [48] and heavy rainfall [39], or predation of the released pupae [40] are the most important abiotic and biotic mortality factors, respectively, before female emergence. In addition, releasing more parasitoids does not necessarily increase parasitism but could reduce parasitism levels recorded in the field [51]. Most biocontrol agents released in ABC programs have an optimal release rate that produces effective control of host pest [52], highlighting the need for better-defined release protocols for *T. remus*. Furthermore, the best biological control practices should not only aim to increase biological control efficacy but also mitigate any risks associated with its careless adoption [53].

Therefore, the present study aimed to improve the recommendations for *T. remus* releases. To enhance the release of another egg parasitoid of the same genus, *Telenomus podisi* Ashmead (Hymenoptera: Scelionidae), which parasitizes eggs of stink bugs (Hemiptera: Pentatomidae), previous work successfully developed a honey-solid diet formed by 100% honey soaked in 100%-cotton strings (Tex 378, Ne 8/5 Thread, Charm^®^ Natural Circle) for one minute and left to dry for an hour before being offered to the adult parasitoids in small pieces [54]. This honey-solid diet avoided possible parasitoid mortality caused by honey droplets to the fragile wasps, which, due to the viscous nature of the honey, can stick parasitoids, potentially leading to their death [54]. In addition, this solid-honey diet prolonged *T. podisi* storage, allowing the parasitoid to be kept for up to 14 days under environmental temperature (25 °C), after parasitoid emergence, before being released in the field, without any reduction in its parasitism capacity [54]. However, such a honey-solid diet had never been tested for *T. remus*.

Consequently, this work (a) studied the parasitism capacity of *T. remus* fed with the honey-solid diet compared to the traditional honey-droplet diet to verify the suitability of such diets for *T. remus*, (b) evaluated the parasitism capacity of *T. remus* fed with the honey-solid diet and the same parameters, but with parasitoids at different times after emergence to check the effect of storage on the parasitoid quality, and (c) tested the parasitism of several parasitoid densities of *T. remus* adults (from 24 to 48 h) fed with this honey-solid diet, under field conditions. Together, these approaches aimed to optimize both the quality and quantity aspects of *T. remus* deployment, ultimately enhancing its adoption and effectiveness as a biocontrol agent against FAW.

## 2. Materials and Methods

### 2.1. Insect Rearing

Eggs of *S. frugiperda* as well as *T. remus* females used in the experiments were from insect colonies maintained in laboratory conditions [25 ± 2 °C, 80 ± 10% RH, and 14 h:10 h Light:Dark (L/D) photoperiod].

*S. frugiperda* specimens were originally collected from maize plants (C-strain) at the Embrapa Field Station, Londrina, State of Paraná, Brazil (23°21′19.2″ S, 51°10′16.8″ W), in 2020 and morphologically identified using the Manual for the Identification of Insects and Other Invertebrates of Soybean Crops [55]. Since then, the insects have been maintained in the rearing, during which new field insects were introduced each year to maintain the quality of the colony.

Larvae of *S. frugiperda* were initially individualized in plastic cups (50 mL) containing an artificial bean-based diet [56] until reaching the pupae stage. Then, pupae were separated by sex [57] and transferred to acrylic cages (45 × 33 × 35 cm) for eclosion of the adults and their mating and oviposition. The adults were fed a diet based on honey (10%) and distilled water. The cage was covered internally with white paper as oviposition substrate, and the eggs were collected daily for experiments or colony maintenance.

*T. remus* was originally collected in Ecuador and reared at the parasitoid rearing facilities of ESALQ/USP (Luiz de Queiroz College of Agriculture, University of São Paulo). In 2008, a subset of this population was transferred to Embrapa Soybean, where it has since been maintained using individuals exhibiting favorable biological traits. Since then, *T. remus* has been reared in the laboratory using *S. frugiperda* egg masses (approximately 150 eggs each), which were glued onto cards (2 cm × 8 cm) and introduced into tubes together with eggs previously parasitized by *T. remus*. Small drops of honey were placed inside these tubes to feed the adults as soon as they emerged. The tubes were then closed, and the eggs allowed to be parasitized for 24 h. Adults that emerged from these eggs were used for trials or colony maintenance.

### 2.2. Experiment 1: Parasitism Capacity of Telenomus remus Fed on Liquid (Honey-Droplet Diet) vs. Honey-Solid Diet

The experiment was carried out in climatized chambers (ELETROLab^®^, model EL 212, São Paulo, SP, Brazil) at temperature of 25 ± 2 °C, 80 ± 10% of relative humidity, and 14 h:10 h Light:Dark (L/D) photoperiod in a completely randomized design with two treatments (diets) and 20 replicates (each one composed by an individualized female ≤48 h with the tested diet). The studied diets were: (1) 100% honey offered in tiny droplets applied to the tube walls every two days (honey-droplet diet) provided ad libitum, and (2) 100% honey soaked into cotton strings (honey-solid diet) as proposed for *T. podisi* [54].

Different tubes containing *T. remus* pupae from the parasitoid rearing received one of the tested diets 6 days before emergence for feeding the recently emerged adults. Then, 48 h after emergence of the first adults, parasitoid females (20 females for each treatment) were individualized in Duran acrylic tubes (6 cm high and 1 cm in diameter) with the tested diet. Each *T. remus* female (replicate) was provided a card (1.0 × 0.7 cm) with approximately 150 *S. frugiperda* eggs (<24 h old), which were replaced daily until the parasitoid’s death. The evaluated biological parameters included: (1) daily parasitism (number of eggs parasitized per day), (2) lifetime parasitism (total number of eggs parasitized per female during its lifetime), (3) emergence rate (%), (4) sex ratio (proportion of females), calculated as sr = number of females/(number of females + number of males), and (5) longevity of parental females (measured in days after release, with “release” defined in this experiment as the day parasitoids first received host eggs).

### 2.3. Experiment 2: Shelf Life of Telenomus remus Adults Inside Capsules with Honey-Solid Diet

The experiment was carried out in the same climatized chambers and controlled conditions previously described for experiment 1, in a completely randomized design with 4 treatments (different storage periods of *T. remus* inside capsules with honey-solid diet at 25 °C) and 20 replicates formed by individualized females with the solid diet. The evaluated periods of storage (treatments) were 2 (control), 4, 6, and 8 days after the emergence of the first *T. remus* adults inside the capsules (males). A commercial, spherical capsule made of cellulose (Agribela Tecnologias Biológicas^®^, Bandeirantes, PR, Brazil) with a 3.0 cm diameter was used, containing 150 pupae of *T. remus*.

A total of 16 capsules were prepared 6 days before the emergence of the first *T. remus* adults and stored inside the same climatized chambers (ELETROLab^®^, model EL 212, São Paulo, SP, Brazil). Every 24 h, the capsules were handled (turned over) to simulate possible shaking during the transport of the capsules from the production facility (biofactory) to the field. On each evaluation day (treatments), 4 capsules were opened and evaluated for the number of living and dead adult parasitoids inside each capsule. In addition, 20 females from the opened capsules (20 replicates) were selected to perform a parasitism capacity trial. Each replicate was composed of an individual female *T. remus* in a Duran acrylic tube (height 6 cm, diameter 1 cm) with white cards (1.0 × 0.7 cm) containing 150 eggs (≤24 h old) of *S. frugiperda*, which were replaced daily until the death of the female. The honey droplets were provided in the tube for feeding the adults.

The following biological variables were evaluated: daily (number of eggs parasitized per day) and lifetime parasitism (total number of eggs parasitized per female), emergence (%), sex ratio (female proportion) [sr = number of females/(number of females + number of males)], parental longevity after release (number of days the parasitoid survived after opening the capsules), and number of living and dead adult parasitoids inside each opened capsule.

### 2.4. Experiment 3: Field Performance of Telenomus remus Fed Honey-Solid Under Different Release Densities

**Figure 1:**
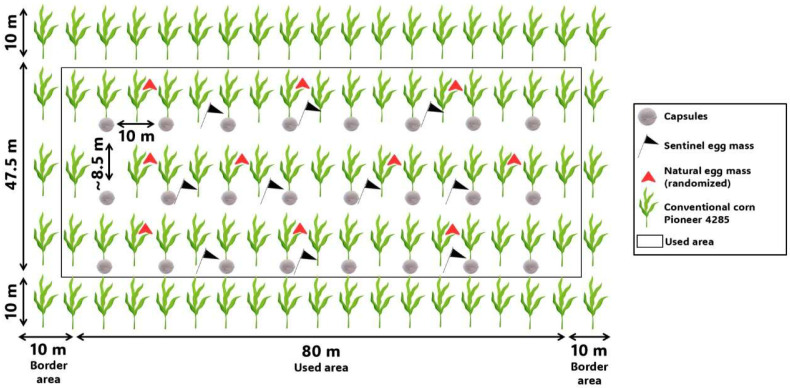
Representation of each pseudo-replication adopted in the release field trial of *Telenomus remus* fed on honey-solid carried out in Londrina, Paraná, Brazil, in maize.

The experiment was carried out in field conditions (23°21′19.2″ S, 51°10′16.8″ W) in a maize field measuring 270 × 400 m at the Brazilian Research and Agricultural Corporation (Embrapa Soja), in Londrina, PR, Brazil, during the 2024 maize growing season. The experimental area was divided into four field plots (270 × 100 m, totaling 2.7 ha), each assigned to one *T. remus* release density (treatment). Within each treatment plot, four subsampling points (pseudo-replications) were used to evaluate parasitism (67.5 × 100 m). In each pseudo-replication (Figure 1). A border area of 10 m was excluded in the evaluation, and a used area of 47.5 × 80 m was adopted for *T. remus* release and sampling of *S. frugiperda* eggs (for parasitism evaluation).

Treatments consisted of different release densities of *T. remus* adults fed the honey-solid diet previously described [40] as follows: (i) 5000; (ii) 10,000; (iii) 15,000; and (iv) 20,000 parasitoids per hectare. All parasitoids were obtained from the *T. remus* colony maintained at the Parasitoid Laboratory of Embrapa Soybean, where they were reared on *S. frugiperda* eggs under controlled conditions. Sentinel egg masses used for parasitism evaluation originated from the same laboratory colony of *S. frugiperda*.

A baseline assessment was carried out before the first parasitoid release to evaluate the presence of natural parasitism in the area. Subsequently, parasitoids were released 3 times for each treatment plot, spaced from seven to ten days apart each release. Parasitoids were released as mated and honey-solid-fed adults, with 24–48 h post-emergence, ensuring that they were physiologically ready for host searching and parasitism. Adults were released inside biodegradable capsules, which were opened in the field at the time of release to allow the parasitoid dispersion. Capsules were evenly distributed across the field, with 35 release points per hectare [21]. In treatments with higher release densities, the number of capsules per hectare was proportionally increased, but the number of releasing points was kept the same (35 points/ha) to maintain homogeneous distribution and prevent capsules from being overcrowded.

Parasitism evaluations were conducted at 1, 2, and 6 days after each release (at 24-, 48-, and 144 h post-release). In each sampling, 10 naturally laid egg masses and 10 sentinel egg masses were collected per replicate in the area of each pseudo-replication (Figure 1). Sentinel eggs consisted of laboratory-produced *S. frugiperda* eggs glued onto cardboard strips (2.5 × 5 cm) and fixed on a flag in the field. All collected egg masses were individually transferred to plastic vials and brought to the entomology laboratory of Embrapa Soybean, where they were kept in climate chambers (ELETROLab^®^, model EL 212, São Paulo, SP, Brazil, regulated at 25 ± 2 °C, 80 ± 10% RH, 14:10 h L:D photoperiod). Egg masses were monitored daily to record parasitoid emergence. Emerging *S. frugiperda* larvae were immediately removed to avoid cannibalism of parasitized eggs. Two response variables were analyzed: parasitism rate and proportion of parasitism between egg masses (natural and sentinel) per sampling date.

### 2.5. Statistical Analysis

Parasitism rate was analyzed using generalized linear mixed models (GLMMs) with a binomial error distribution and a logit link function, fitted with the glmmTMB package [58]. The model included treatment, sampling date, their interaction as fixed effects, and block as a random effect. When the interaction was significant, we performed pairwise comparisons of treatments within each date using Tukey’s test (*p* < 0.05) via the emmeans package. Emergence rate (%) and sex ratio (proportion of females) were analyzed using generalized linear models (GLMs) with binomial error distribution and logit link function. Adult female longevity data were analyzed using one-way analysis of variance (ANOVA) to assess the effects of diet and storage duration on survival. When significant differences were detected, means were compared using Tukey’s test (α = 0.05). For GLM and GLMM, estimated marginal means and pairwise comparisons were calculated using the emmeans package [59], with Tukey adjustment for multiple comparisons where appropriate, also using the emmeans package. All statistical analyses were performed in R version 4.3.2 [60].

## 3. Results

### 3.1. Experiment 1: Parasitism Capacity of Telenomus remus Fed on Liquid (Honey-Droplet Diet) vs. Honey-Solid Diet

Overall, better parasitoid performance was recorded for adult parasitoids that received the honey-droplet diet. Although the difference was statistically significant, the lifetime number of *S. frugiperda* eggs parasitized was only 15.34% higher in parasitoids fed with this diet (165.4 eggs) compared to those fed with the honey-solid diet (143.4 eggs) (χ^2^ = 914.21, df = 13, *p* < 0.0002, Table 1).

Significant differences in the emergence (%) of parasitoids were also observed between diets (χ^2^ = 44.51; df = 1; *p* < 0.001), but again with only 4% difference. We recorded 77.7% emergence (F2) from parasitism from adults fed with the honey-droplet diet compared to 73.7% emergence (F2) from parasitism from adults fed with the honey-solid diet. However, no difference among *T. remus* diets was recorded for the progeny sex ratio (χ^2^ = 0.0023; df = 2; *p* < 0.724) or the parental longevity of adult females (days) (F_(1, 8)_ = 1.01; *p* < 0.300), which were not influenced by the tested diets (Table 1).

**Figure 2:**
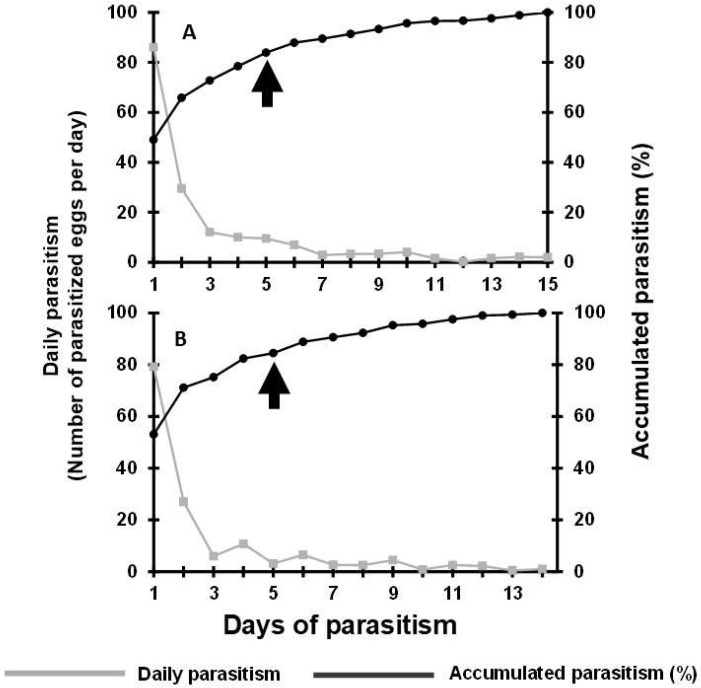
Parasitism capacity of *Telenomus remus* fed with honey-solid diet (**A**) and honey-droplet diet (**B**) on eggs of *Spodoptera frugiperda*. Arrows indicate 80% of total parasitism (accumulated parasitism%).

Numerically, the highest number of parasitized eggs per day was recorded on the first day of parasitism, and 80% of total parasitism (accumulated parasitism%) was reached by the fifth day of parasitism for parasitoids reared on both tested diets (honey-solid and honey-droplet diets) (Figure 2). In the first 24 h of parasitism, *T. remus* fed with the honey-solid diet parasitized an average of 86.1 eggs (Figure 2A), while the parasitoids fed with the honey-droplet diet parasitized 79.2 eggs (Figure 2B).

### 3.2. Experiment 2: Shelf Life (Storage Period in Days) of Telenomus remus Adults Inside Capsules with a Honey-Solid Diet

**Figure 3:**
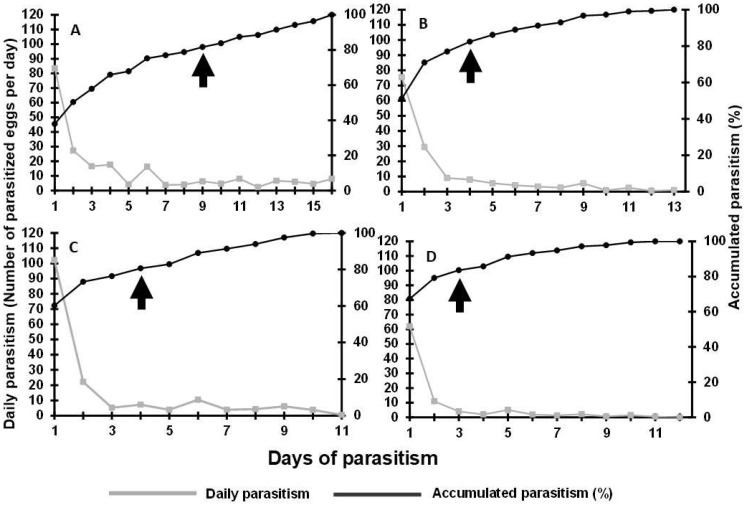
Parasitism capacity of *Telenomus remus* fed with a honey-solid diet at different periods after parasitoid emergence. (**A**) two days, (**B**) four days, (**C**) six days, (**D**) eight days after adult parasitoid emergence. The arrows indicate 80% parasitism.

The highest number of parasitized *S. frugiperda* eggs was observed during the first 24 h of parasitism (83.4, 75.5, 102.2, and 62.0 eggs) by *T. remus* stored for 2, 4, 6, and 8 days, respectively (Figure 3). We recorded 80% parasitism at 9 days of parasitism (*T. remus* females stored ≤ 2 days) (Figure 3A), 4 days of parasitism (*T. remus* females stored ≤ 4 days) (Figure 3B), 4 days of parasitism (*T. remus* females stored ≤ 6 days) (Figure 3C), and only 3 days of parasitism (*T. remus* females stored ≤ 8 days) (Figure 3D). Thus, 80% parasitism was reached faster after the longest storage periods.

The lifetime number of eggs parasitized was significantly influenced by the storage periods, decreasing as storage time increased, with no differences among 4 and 6 days of storage (χ^2^ = 773.41; df = 3; *p* < 0.002). The highest number of eggs parasitized during the females’ lifetime occurred in the shortest storage period, 193.4 (storage ≤ 2 days), followed by 150.4 (storage ≤ 6 days) and 143.6 (storage ≤ 4 days). The lowest lifetime number of parasitized eggs, 86.6, was recorded for females stored for 8 days (Table 2). This showed that the longest storage time had a marked impact on the parasitism capacity of *T. remus*, with a reduction of 42.4% in the parasitism at eight days of storage compared to six days of storage.

Despite statistically significant differences among some treatments, emergence rates were not only greater than 74% for all evaluated periods of storage (treatments) but also varied only slightly among treatments, ranging from 74.4% to 80.5% (χ^2^ = 53.6; df = 3; *p* < 0.001), and the longevity of parental adult females ranged from 7.1 to 11.5 days [F(3, 27) = 33.07; *p* < 0.001], without exhibiting any clear trend. The emergence of the progeny of *T. remus* stored for 2 and 8 days was statistically similar. Likewise, the results show progeny sex ratio ranging from 0.56 to 0.80 (Deviance = 63, df = 3, *p* < 0.006).

The evaluation of parasitoids stored during the opening of the capsules revealed similar emergence rates of the adults placed inside the capsules 6 days before emergence, together with the honey-solid diet, regardless of the storage duration, and overall low mortality. The percentage of dead parasitoid adults found inside the capsules ranged from 2.1% (2 days post-emergence) to 5.9% of the total adults per capsule (6 days post-emergence), with significant differences among treatments (Table 3). However, this difference was mainly driven by the lower mortality observed in the 2-day group, which differed significantly from the others. No significant differences were found among the remaining treatments. Moreover, the mortality rate of the emerged adults was always below 6%.

### 3.3. Experiment 3: Field Performance of Telenomus remus Fed Honey-Solid Under Different Release Densities

**Figure 4:**
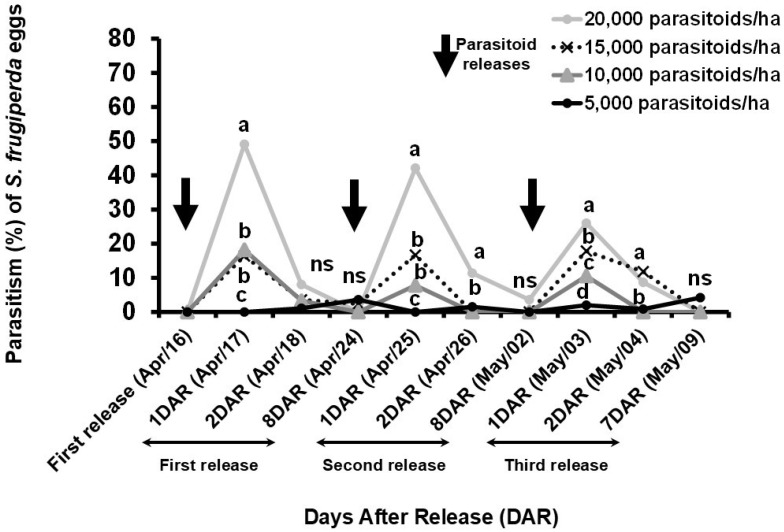
Release of different densities of *Telenomus remus* (parasitoids/ha) in maize during the second cropping season of 2024, conducted at the Embrapa Soja experimental field (Londrina, Paraná, Brazil) (experiment 3). Letters indicate significant differences among treatments on each date, based on generalized linear mixed models with binomial distribution and logit link, followed by pairwise comparisons using Tukey’s test on estimated marginal means (*p* < 0.05). Non-significant results were indicated as “ns” (*p* ≥ 0.05). Arrows indicate when the parasitoid was released.

The field parasitism rate of *S. frugiperda* eggs recorded after the releases of different densities of *T. remus* positively responded to the increase in numbers of parasitoids (χ^2^ = 3616.1, df = 3, *p* < 0.001). Overall, parasitism rates were higher in the treatment of 20,000 parasitoids/ha than parasitism recorded with 15,000 parasitoids/ha and 10,000 parasitoids/ha (χ^2^ = 62.74, df = 3, *p* < 0.001), while parasitism of 10,000 and 15,000 parasitoids/ha did not differ between themselves (χ^2^ = 5.82, df = 3, *p* = 0.12). Furthermore, the highest parasitism was recorded in the treatment with 20,000 parasitoids/ha, reaching a peak of 49.1 ± 6.1% 24 h after the first release (Figure 4). The highest parasitism observed in the 15,000/ha treatment was 17.9 ± 2.6%, which, despite being similar to the parasitoid release density of the 20,000 parasitoids/ha treatment, was substantially lower in effectiveness. In contrast, the 5000 parasitoids/ha treatment consistently showed the lowest parasitism levels, often near zero throughout the entire evaluation period.

In all treatments, parasitism consistently peaked 24 h after each release and dropped sharply afterward. In the first release, the parasitism rate in the 20,000/ha treatment fell from 49.1% to only 8.0% on the second day. In the second release, the decline was from 42.1% to 11.0 ± 1.6%.

**Figure 5:**
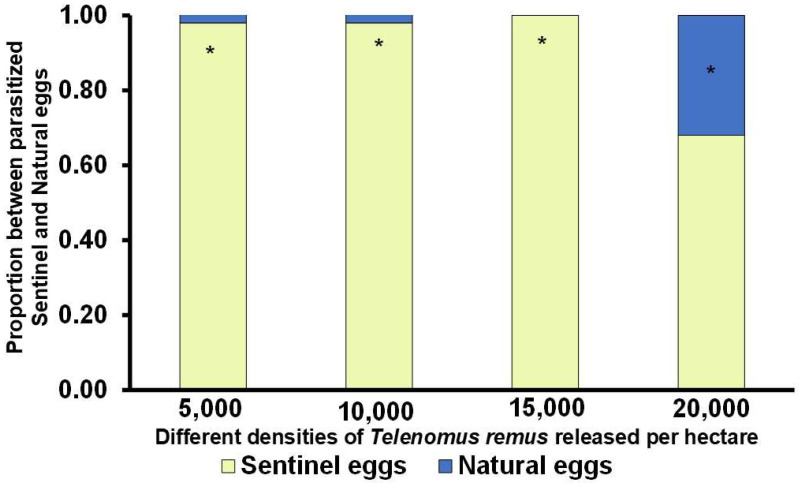
Proportion of parasitized sentinel and natural eggs of *Spodoptera frugiperda* (Lepidoptera: Noctuidae), following releases of *Telenomus remus* (Hymenoptera: Scelionidae) at different densities in maize during the second cropping season of 2024. The experiment was conducted at the Embrapa Soja experimental field, Londrina, Paraná, Brazil. * Represent parasitoid densities with statistical difference between the proportion of sentinel eggs and natural eggs based on generalized linear mixed models with binomial distribution and logit link, followed by pairwise comparisons using Tukey’s test on estimated marginal means (*p* < 0.05).

Nearly all parasitism recorded in the 5000, 10,000, and 15,000 parasitoids/ha treatments occurred in sentinel egg masses (Figure 5). In contrast, parasitism in the 20,000/ha treatment occurred approximately in a 70%:30% ratio between sentinel and natural egg masses, indicating a broader dispersal and activity of the parasitoids at higher release densities.

Parasitism trends over time clearly highlight the superior performance of the 20,000 parasitoids/ha treatment, with pronounced peaks and greater temporal impact. Lower-density treatments maintained minimal and stable parasitism rates throughout the study period.

## 4. Discussion

Overall, the reported results illustrate that the release of fed adult parasitoids can be adopted using the tested honey-solid diet previously described in the literature [54]. Being able to release *T. remus* as fed adults could increase parasitoid survival after release compared to the most common strategy currently adopted of releasing egg parasitoid pupae in bulk [34,54,61]. The survival of parasitoids released as pupae can be reduced by predation [40], temperature [62], and rainfall [39], among other causes of mortality. This is especially true for scelionids (Hymenoptera: Scelionidae), whose males emerge up to 24 h before females, leading to female pupae being exposed for longer periods in the field to the different causes of mortality [63]. When exposing parasitoid pupae of another *Telenomus* species (*T. podisi*) in soybean fields for only 24 h, a previous study found a significant reduction in adult emergence from 76% (control) to close to 20% when pupae were directly exposed to sunlight between soybean rows (hotter spots) [62].

Furthermore, our results indicate that *T. remus* fed with the honey-solid diet can be stored up to 6 days before release with low reduction in lifetime parasitism, which can be compensated for by increasing the number of released parasitoids in the field by around 30%. In addition, the recorded sex ratio was always higher than 0.50, which is similar to previously recorded results [18,64]. Having a similar or higher proportion of *T. remus* females in the parasitoid population is important since they are responsible for the parasitism [18].

Unlike storing *T. remus* for up to 6 days, 8 days of parasitoid storage should be avoided due to the significant reduction in both the lifetime number of parasitized eggs per female and the parental longevity of females to only 7.1 days. Although storage of 6 days is apparently short, prolonging the shelf life of the *T. remus* bioinsecticide from zero to 6 days is a significant advancement that could prove beneficial in cases of rainy or extremely hot days, or even short delays in the transport of the parasitoid from production sites to the field, while still preserving the efficacy of the parasitoid [54].

Thus, the possibility of using the honey-solid diet to feed *T. remus* adults is an important innovation for the success of the parasitoid release. As a synovigenic parasitoid, *T. remus* females store few to no mature oocytes in their abdomen at the time of emergence [54,65,66]. Thus, parasitoid adult females continue to produce and mature eggs throughout adult life, requiring them to regularly acquire nutrients for egg production [67]. Therefore, fed *T. remus* can minimize food foraging efforts, focusing parasitoid energy into locating and parasitizing its hosts. Additionally, parasitoids with access to sugar sources in the field have longer lifespans and higher parasitism rates than those experiencing food deprivation [68]. Once depleted of mature eggs, fed parasitoid females have also been reported to contribute to greater non-reproductive host mortality [69]. However, nectar or honeydew, the most common natural food sources for parasitoids, are usually scarce in large commodity crops such as maize [68,70]. Low availability and accessibility of food sources for parasitoids strongly reduce parasitoid retention in the field and host-finding efficacy [71].

In light of these physiological and ecological constraints, providing immediate shelter to the parasitoids can likely enhance their initial efficacy, since parasitism rates at the field level were consistently highest within the first 24 h post-release. Previous results published in the scientific literature also reported *T. remus* parasitism peaking on the first day [72] and starving parasitoids tend to prioritize foraging over host hunting [66].

Despite the high potential for technological innovation brought by the release of fed *T. remus* adults, field parasitism is also strongly tied to the right parasitoid release density [41]. Biological IPM programs with *T. remus,* with releases at the density of 20,000 parasitoids/ha, will probably require additional control strategies to keep *S. frugiperda* under the economic injury level since *T. remus* parasitism in the field did not reach 80% of the eggs. This release density is higher than the previous proposal of 5000 to 10,000 wasps per hectare per season [18] and previously successfully adopted in Venezuela during the 1990s [21,42,44]. On the other hand, 20,000 parasitoids/ha is lower than the release density proposed in the *T. remus* registration in Brazil, which occurred in July 2024, suggesting 3 releases of 40,000 parasitoids/ha [73]. Those divergent results indicate that future field trials should evaluate higher densities of *T. remus*. Less expensive massive rearing protocols still need to be developed to make this higher release density commercially viable.

Our trial was carried out in the second season (autumn/ winter), whereas most of the previous published trials were performed in the first crop season of maize (summer season), which has very different weather conditions. Although some field trials have been conducted in Brazil, only one study has reported successful parasitism rates above 70%. This exception involved the release of 90,000 to 120,000 parasitoids/ha, achieving rates of 72.4% and 82.8% [45], a significantly higher density than the 20,000/ha tested in our field trial. Despite higher *T. remus* release density being limited by the current costs involved in rearing of its hosts [18,74], artificial eggs are being intensively studied [75]. The potential of artificial rearing technologies could revolutionize biological control, reducing the reliance of agriculture on chemical pesticides, emphasizing the role of biological control as a cornerstone of modern sustainable agriculture [76].

A combination of biological and ecological factors may explain the limited parasitism observed in natural egg masses at lower release densities. First, sentinel egg masses were placed in accessible and exposed positions, greatly facilitating host detection, despite its great parasitoid dispersion capacity [21]. In contrast, natural *S. frugiperda* eggs are often laid in concealed locations, such as between the base of the leaves of maize plants [77], where physical access is more difficult for the parasitoid. Second, *T. remus* exhibits a narrow host age window, with the highest parasitism success occurring in eggs 24 to 48 h after release, and negligible parasitism beyond 72 h after release [78]. Thus, some of the natural eggs present at the time of parasitoid release may have been too old to be parasitized. Finally, the increase in parasitism of natural egg masses observed only at the highest release density can indicate a clear density-dependent effect. This pattern is consistent with previous reports [22,45,79], which also describe a high number of active females needed per egg mass to ensure sufficient parasitoid coverage, reaching up to 45 *T. remus* females per 300 *S. frugiperda* eggs. Such coverage is essential to overcome spatial and temporal limitations that often occur under field conditions and may vary depending on the structure and phenology of the crop.

The release intervals of 7 to 10 days may not be sufficient to maintain adequate control pressure, particularly under conditions of high infestation or pest migration. A higher number of releases at shorter intervals extends the period in which active parasitoid populations are present in the field, thereby reducing the risk of pest resurgence from either survivors or new incoming individuals [42]. Therefore, future studies should consider evaluating strategies that involve more frequent releases and/or higher parasitoid densities, aiming to enhance the overall effectiveness of biological control and to ensure longer-lasting protection over vulnerable pest stages.

## 5. Conclusions

The storage period after the emergence of the first adult *Telenomus remus* can be carried out up to 6 days after parasitoid emergence using the studied honey-solid diet, making the release process of *T. remus* more flexible and attractive to farmers. This increased shelf life could be extremely helpful when the release of *T. remus* must be delayed for one or two days, due to bad weather or other reasons. In addition, the release of fed adults should also reduce predation and other causes of mortality to which immobile *T. remus* pupae are more susceptible than adults.

The release density of 20,000 parasitoids per hectare seems to be the most effective among the studied rates (from 5000 to 20,000) and could be recommended as part of an integrated pest management (IPM) strategy, given that it resulted in approximately 50% control of *Spodoptera frugiperda* egg masses under field conditions, considering the second season (autumn/winter) in Brazil. However, this level of suppression may still be insufficient as a stand-alone strategy. Therefore, future studies should aim to evaluate the efficacy of higher *T. remus* release densities, perform cost-effectiveness analyses, and explore shorter release intervals to optimize the practical use of egg parasitoids in field conditions. In addition, a cheaper mass production protocol for *T. remus* is important to study for higher than 20,000 parasitoids/ha to be economically feasible in field conditions.

## Figures and Tables

**Table 1:** *Telenomus remus* parasitism capacity on eggs of *Spodoptera frugiperda* with adult parasitoids feeding on different diets.

Diet	Lifetime Number of Parasitized Eggs/Female ^1^	Emergence (%) ^2^	Progeny Sex Ratio ^2^	Parental Longevity of Adult Females (Days) ^3^
100% honey in tiny droplets (honey-droplet diet)	165.4 ± 5.88 a	77.7 ± 1.84 a	0.69 ± 0.04 a	10.4 ± 0.71 a
100% honey in macerated cotton strings (honey-solid diet)	143.4 ± 4.57 b	73.7 ± 1.01 b	0.70 ± 0.02 a	10.1 ± 0.40 a

Means ± Standard Error (N = 64) followed by the same letter within columns did not statistically differ from each other by ^1^ (GLMM *p* > 0.05), ^2^ (GLM *p* > 0.05), and ^3^ (ANOVA *p* > 0.05).

**Table 2:** *Telenomus remus* parasitism capacity on eggs of *Spodoptera frugiperda* on different days after emergence, stored inside release capsules with a honey-solid diet.

Days of storage After Parasitoid Emergence	Lifetime Number of Parasitized Eggs/Female ^1^	Emergence (%) ^2^	Progeny Sex Ratio ^2^	Parental Longevity of Adult Females (Days) ^3^
2	193.4 ± 8.82 a	79.9 ± 1.78 a	0.74 ± 0.02 a	11.5 ± 0.63 a
4	143.6 ± 4.35 b	74.4 ± 1.75 b	0.80 ± 0.01 a	10.0 ± 0.35 a
6	150.4 ± 14.79 b	76.5 ± 1.79 b	0.56 ± 0.04 b	7.4 ± 0.69 b
8	86.6 ± 5.63 c	80.5 ± 1.88 a	0.71 ± 0.08 a	7.1 ± 0.54 b

Means ± Standard Error followed by the same letter within columns did not statistically differ from each other by ^1^ (GLMM *p* > 0.05), ^2^ (GLM *p* > 0.05), and ^3^ (Log rank test *p* > 0.05).

**Table 3:** *Telenomus remus* emergence (%) and dead adults (%) trapped inside capsules on different days after adult emergence from *Spodoptera frugiperda* eggs, with parasitoid adults feeding on a honey-solid diet.

Days After Parasitoid Emergence	Emergence (%) of Adults from Pupae Inside Capsules	Dead Adults (%) Trapped Inside Capsules
2	69.8 ± 2.76 a	2.1 ± 0.61 a
4	74.6 ± 2.56 a	5.2 ± 0.50 b
6	73.3 ± 3.58 a	5.9 ± 1.03 b
8	75.0 ± 0.97 a	5.5 ± 0.80 b

Means ± Standard Error followed by the same letter within columns did not statistically differ from each other (GLM *p* > 0.05).

## Data Availability

Raw data available in the Supplementary Materials.

## Supplementary Materials

The following supporting information can be downloaded at: https://www.mdpi.com/article/10.3390/insects16101032/s1, Table S1: Raw data from experiment 1 [Lifetime number of parasitized eggs/female; number of emerged parastioid; parental longevity (days); sex ratio], Table S2: Raw data from experiment 2 [Lifetime number of parasitized eggs/female; number of emerged parastioid; parental longevity (days); sex ratio; number of dead adults inside capsules], Table S3: Raw data from experiment 3 [egg mass type (sentinel or natural), total number of eggs, number of eggs parasitized].
